# Self-video modeling combined with self-feedback in youth sport: an opinion on cognitive load, attention, and learning design

**DOI:** 10.3389/fpsyg.2026.1775088

**Published:** 2026-02-26

**Authors:** Amayra Tannoubi, Vlad Adrian Geantă, Vasile Emil Ursu, Fairouz Azaiez

**Affiliations:** 1 High Institute of Sport and Physical Education of Gafsa, University of Gafsa, Gafsa, Tunisia; 2 Sports Performance Optimization Research Laboratory (LR09SEP01), National Center for Sports Medicine and Science (CNMSS), Tunis, Tunisia; 3 Department of Physical Education and Sport, Faculty of Physical Education and Sport, Aurel Vlaicu University of Arad, Arad, Romania; 4 Department of Physical Education and Sport, Faculty of Law and Social Sciences, University “1 Decembrie 1918” of Alba Iulia, Alba Iulia, Romania

**Keywords:** acquisition, ecological model, feedback, learning, video modeling

## Abstract

**Figure:**
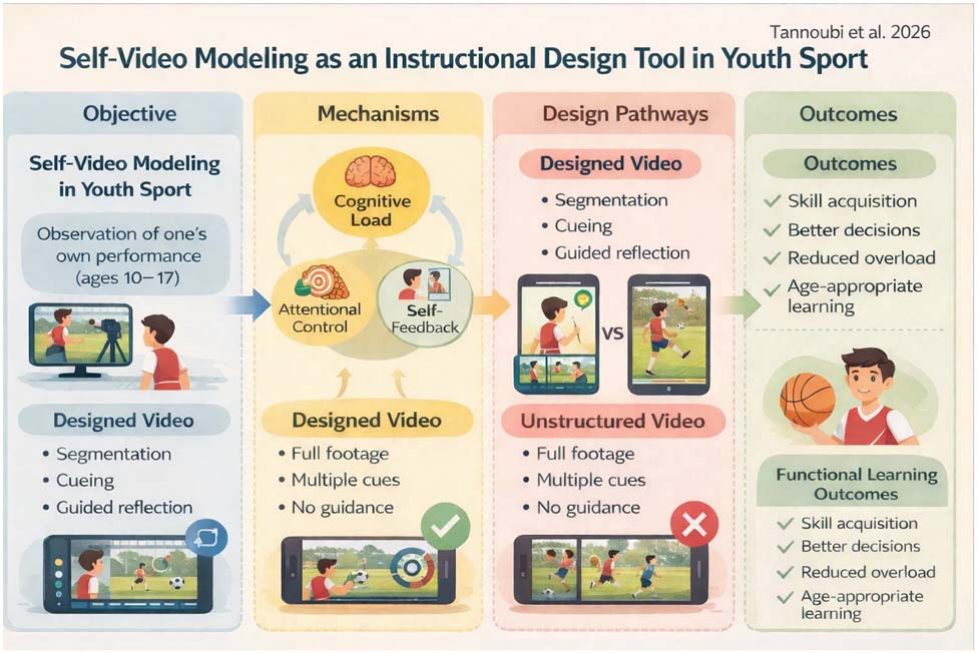
Conceptual framework illustrating self-video modeling as an instructional design tool in youth sport. Structured video features (segmentation, cueing, and guided reflection) support attentional control, self-feedback, and cognitive load regulation, leading to functional learning outcomes compared with unstructured video viewing.

Conceptual framework illustrating self-video modeling as an instructional design tool in youth sport. Structured video features (segmentation, cueing, and guided reflection) support attentional control, self-feedback, and cognitive load regulation, leading to functional learning outcomes compared with unstructured video viewing.

## 1 Introduction

The rapid diffusion of digital technologies in physical education (PE) and youth sport has led to the widespread adoption of video-based learning practices. Among these, self-video modeling is increasingly used under the implicit assumption that visual access to one's own performance is inherently beneficial for learning. In our view, this assumption is conceptually fragile. Learning outcomes are shaped less by instructional media than by the task-relevant psychological processes (e.g., selective attention, metacognitive evaluation) (Hattie and Timperley, 2007; Mayer, 2024; Stull et al., 2021), yet video-based practices in youth sport are often implemented with limited consideration of learners' cognitive and attentional constraints. Physical education (PE) prioritizes structured skill-building, whereas youth sport demands adaptation to competitive, dynamic tasks, self-video modeling fits youth sport by externalizing performance for task-aligned reflection.

This issue is particularly salient in youth populations, whose working memory capacity, attentional control, and self-regulatory skills are still developing (Zimmerman, 2000). Despite this, video is frequently treated as a neutral feedback modality rather than as an instructional design element that actively structures cognitive load and attentional focus. We argue that this prevailing approach contributes to the inconsistent learning/performance and sometimes counterproductive outcomes (e.g., stalled skill transfer, motivation dips) (Ste-Marie et al., 2012) observed in applied youth sport settings.

In this opinion article, we contend that the educational value of self-video modeling lies not in video exposure *per se*, but in how video is designed to regulate cognitive load and guide attention when combined with structured self-feedback. This opinion integrates self-video modeling, structured self-feedback, cognitive load regulation, and attentional control into a unified learning-design argument tailored for youth sports (ages 10–17). Youth athletes face developmental limits in working memory and self-regulation (Zimmerman, 2000), making intentional video design critical to avoid overload and guide focus. Although examples from basketball and other invasion sports are often used to illustrate applied practices, the position advanced here is not sport specific. Instead, it applies broadly to youth learning environments characterized by open, perceptually demanding motor tasks (Abernethy, 1993). Instead of a way to give feedback, we consider self-video modeling as a psychological design tool. Here, we adopt a cognitive-ecological hybrid perspective, which integrates the learner's internal cognitive architecture (e.g., working memory limits) with the external perceptual-motor constraints of the sport environment (e.g., informational variables in open tasks) to inform video-based instructional design.

## 2 Self-video modeling: a misunderstood approach to learn

Self-video modeling involves youth athletes watching videos of their own performances, often focusing on moments of success or improvement. While applicable across age group, it is particularly salient in youth sport (ages 10–17) due to ongoing development in working memory, attentional control, and self-regulatory skills (Zimmerman, 2000). From a social cognitive perspective, self-modeling has been linked to enhanced self-efficacy and observational learning (Bandura, 1986; Dowrick, 2012; Tannoubi et al., 2023; Trabelsi et al., 2025). However, its educational potential is frequently oversimplified. Video externalizes performance, allowing athletes to see themselves from an observer's perspective, a view supported by visual perception research (Ishikura and Inomata, 1995, 1998). This externalization can facilitate task-aligned reflection, yet performance analysis highlights inherent processing challenges due to information density and multi-angle overload (Bampouras et al., 2012).

Self-video modeling is frequently presumed to facilitate learning automatically, regardless of the learners' engagement with or interpretation of the visual content. Evidence from observational learning research suggests otherwise: without attentional guidance, learners may focus on irrelevant movement features, engage in superficial evaluation, or become overwhelmed by visual detail (Ste-Marie et al., 2012). Work on self-confrontation approaches further indicates that structured confrontation with one's own performance can promote reflective awareness and meaningful performance interpretation when appropriately scaffolded, rather than merely exposing learners to visual feedback (Rochat et al., 2019). From our perspective, ineffective outcomes therefore reflect not learner limitations alone, but a failure to recognize that video analysis is cognitively demanding and requires deliberate instructional framing.

Accordingly, the variability of outcomes associated with self-video modeling should not be attributed to inconsistent learner engagement alone, but to inconsistent instructional design. Thus, instructional design must frame video to harness its potential; this sets the stage for structured self-feedback as a complementary mechanisms.

## 3 Self-feedback as an essential—but inadequate—prerequisite

Self-feedback is an essential part of self-regulated learning frameworks, facilitating performance monitoring, reflection, and adaptive modification (Zimmerman, 2000; Zimmerman and Moylan, 2009). When learners actively evaluate their performance against task objectives, such as analyzing shot selection during basketball scrimmages, reviewing defensive positioning in small -sided soccer games, examining run-up rhythm in long-jump training, or assessing stroke timing in swimming drills, they engage metacognitive processes rather than relying solely on external feedback (Hattie and Timperley, 2007; Nicol and Macfarlane-Dick, 2006). For example, a youth soccer player might compare their passing decision in a video clip against a coach's prompt (“Did you choose the best passing option given the defenders' positioning?”), while a gymnast may evaluate body alignment during a vault approach relative to technical cues. These activity-embedded analyses shift evaluation from outcome-focused (“success or failure”) to process-focused appraisal grounded in perception–action coupling and decision quality.

However, we suggest that unguided self-feedback may be problematic for youth learners. Studies show that without instructional scaffolding, self-feedback can become too harsh, unfocused, or not in line with what the task demands (Hadwin et al., 2017; Panadero, 2017). In our view, self-feedback should be treated as a guided process rather than an innate learner capacity. When combined with self-video modeling, structured self-feedback can transform observation into a functional learning cycle linking perception, evaluation, and intention. Yet this combination is only effective if the cognitive and attentional demands imposed by video are explicitly managed. Guided self-feedback alone is insufficient without managing video's cognitive demands, as explained next through cognitive load theory (Sweller, 2011).

## 4 Cognitive load as the central design problem

We argue that cognitive load theory offers the most coherent explanation for the mixed outcomes observed in video-based learning practices in youth sport. The limited capacity of working memory makes learning harder, and instructional materials that add too much extra load are likely to make learning harder, no matter how much information they contain (Sweller et al., 2019). This risk is especially pronounced in video-based instruction. Video can help youth athletes understand task structure, but it can also present extraneous or poorly organized information that overwhelms working memory (Ayres and Ackermans, 2025; Paas et al., 2003). In team sports, for instance, video may contain overlapping player movements and background clutter, increasing extraneous load (Fuster et al., 2021; Khacharem et al., 2014). In individual sports, irrelevant kinematic details (e.g., non-critical joint angles) may distract from key technical cues (Abdelkafi et al., 2025; Herrebrøden et al., 2023). In both contexts, disorganized presentation, such as showing full-game footage without segmentation, impedes schema construction without supporting germane processing.

From this viewpoint, self-video modeling should not be perceived chiefly as a feedback instrument, but rather as a cognitive design tool that influences the allocation of limited cognitive resources by learners (Chen et al., 2018). In youth sport context, this manifests through germane load mechanisms guided reflection on segmented clips yields performance gains (e.g., 15% decision accuracy gains post-video) (García-González et al., 2014); linking reduced extraneous load to superior skill execution under dynamics task constraints. When paired with structured self-feedback, video can constrain attentional focus, reduce extraneous cognitive load, and support germane processing. When unstructured, it is likely to increase cognitive load and undermine both learning efficiency and reflective engagement.

In our opinion, failing to recognize this distinction represents a central weakness in current video-based pedagogical practices. This applies specifically to youth athletes navigating perceptually rich sport environments. Effective load regulation requires stabilizing attention, addressed in the following section.

## 5 Beyond self-regulation: attentional control and perceptual anchoring

Discussions of self-video modeling have tended to emphasize self-regulation while largely neglecting attentional control as a distinct psychological mechanism. Yet effective skill learning in open motor tasks depends fundamentally on learners' ability to selectively attend to task-relevant information in perceptually rich environments (Abernethy, 1993; Vickers, 2016).

Youth learners frequently display diffuse attentional focus and inefficient visual search strategies, which can compromise learning even in the presence of accurate feedback. We propose that self-video modeling can function as a perceptual anchor by externalizing key informational variables and stabilizing attentional focus when observation is guided through structured self-feedback.

This position aligns with research indicating that learning is shaped by attentional constraints embedded within instructional design, not solely by conscious regulation. Ecological approaches have contributed substantially by emphasizing perception–action coupling and environmental information as drivers of skill adaptation (Chow et al., 2021; Renshaw et al., 2019). However, their traditional formulations tend to underrepresent the role of internal cognitive processes such as working-memory limitations, attentional capacity, and metacognitive evaluation. Recent scholarship argues not for rejecting ecological perspectives, but for adopting a pragmatic synthesis acknowledging that cognition and environmental constraints jointly shape behavior in applied settings (Collins et al., 2025). Within video-supported learning, this synthesis is particularly necessary: instructional effectiveness depends on simultaneously managing perceptual information and learners' cognitive architecture. Recognizing this dual influence strengthens the applied relevance of hybrid design frameworks by positioning ecological and cognitive perspectives as complementary rather than competing explanatory lenses.

## 6 Design principles and implications

**Figure 1:**
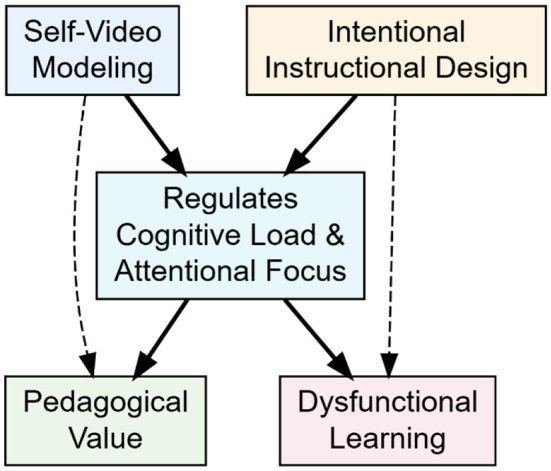
Heuristic framework contrasting intentional video design (functional pathway) vs. unstructured video exposure (dysfunctional pathway) in youth sport learning.

The preceding sections converge on a central thesis: the learning value of self-video modeling is unlocked not by the technology itself, but by instructional designs that deliberately manage cognitive and attentional demands. Translating this thesis into practice requires a shift from using video as a feedback delivery tool to treating it as a learning design problem. Below, we distill our argument into core design principles. These principles are intended not as a prescriptive coaching manual—the development of which would require sport-specific, age-tailored curricula—but as essential criteria to guide the creation of training protocols, digital tools, and coach education programs. The heuristic framework in Figure 1 serves as a visual summary of this design-oriented philosophy, distinguishing between functional (designed) and dysfunctional (unstructured) video applications.

### 6.1 Guiding principle 1: design must be adapted to the learner and task

Effective video-based learning is not one-size-fits-all. Intentional design requires differentiation based on developmental and task constraints, informed by developmental psychology (Zimmerman, 2000) and skill-adaptive learning frameworks (Chow et al., 2021):

- By age: younger athletes (e.g., under 10) benefit from single, visually cued focuses (e.g., “watch knee bend”); older youth (e.g., 15–17) can process multi-element prompts combining visual and verbal guidance.
- By skill level: novices require high levels of support (one goal, modeled feedback); intermediates can integrate two related goals; advanced athletes benefit from structured autonomy in self-analysis.
- By task type: closed skills (e.g., free throw) allow for static clip analysis; open skills (e.g., soccer defending) require dynamic segmentation that preserves decision-making context (García-González et al., 2014).

### 6.2 Guiding principle 2: design must structure the observation and reflection process

To regulate cognitive load and focus attention, video implementation should incorporate the following strategic constraints supported by cognitive load theory (Sweller, 2011) and multimedia learning principles (Mayer, 2024):

- Segment: use short clips (10–20 s) with a single task focus (e.g., dribble footwork only).
- Signal: provide one clear attentional cue per viewing (e.g., overlay text: “Check elbow position”).
- Structure reflection: guide self-feedback with simple, consistent prompts (e.g., Strength: [specific action]; Improvement: [goal-linked change]).
- Control exposure: limit replays to two viewings and incorporate before/after comparison clips where possible.

These principles underscore that the coach's or designer's role is to orchestrate the learning conditions, not merely to provide video access. The significant gap in instructional design knowledge among practitioners, astutely noted in commentary on this work, highlights a critical need: coach education must move beyond teaching how to use video equipment to how to design video-based learning experiences grounded in the cognitive and attentional principles argued here. Figure 1 encapsulates this necessary shift in perspective.

## 7 An opinionated conceptual framework

This opinion is synthesized in the heuristic framework presented in Figure 1. The framework serves to visually articulate our central argument: self-video modeling leads to functional learning outcomes only when it is subsumed within an intentional instructional design process (left pathway). This process, involving mechanisms like cueing, chunking, and prompted reflection, actively regulates cognitive load and stabilizes attentional focus. In contrast, unstructured video exposure (right pathway) represents a common but dysfunctional approach that overlooks the learner's psychological constraints. This figure is not a predictive model nor a practical flowchart, but a conceptual tool intended to challenge tacit assumptions and guide future pedagogical thinking and research in youth sport.

## 8 Conclusion

We argue that self-video modeling must shift from a feedback tool to an instructional design strategy explicitly tailored to regulate cognitive load and guide attention in youth athletes. This approach counters current technology-centric practices that often overlook developmental constraints in working memory and self-regulation. The noted gap in coaches' instructional design knowledge reinforces the need for this shift. Moving forward, the focus should be on how video is designed, not whether it “works.” This requires developing differentiated, principles-based supports—such as age- and skill-adapted cueing, segmentation, and reflection prompts—that can be integrated into coach education, digital tools, and sport-specific protocols. Ultimately, bridging cognitive theory and coaching practice is essential. By treating video as a psychological design tool, we can create more effective and developmentally appropriate learning environments in youth sport.
